# Association between social inclusion and mental health among people with disabilities engaged in sports clubs: a cross-sectional survey

**DOI:** 10.3389/fpsyg.2025.1504352

**Published:** 2025-03-07

**Authors:** Sobhi Saeed Al Harthy, Mohammad Ahmed Hammad, Huda Shaaban Awed

**Affiliations:** ^1^Department of Psychology, Faculty of Education, Umm Al-Qura University, Makkah, Saudi Arabia; ^2^Faculty and Leadership Development Center, Assiut University, Asyut, Egypt; ^3^Department of Psychology, Faculty of Education, Assiut University, Asyut, Egypt

**Keywords:** social inclusion, sports, mental health, people with disabilities, a cross-sectional survey

## Abstract

**Objective:**

Participation in sports is known to promote social inclusion and mental health. However, barriers that limit the participation of people with disabilities in sports potentially exclude them from enjoying the social and mental health benefits. This study aimed to assess the level of social inclusion experienced by people with disabilities participating in sports clubs in Saudi Arabia, and to examine its relationship to specific mental health outcomes, namely, overall mental health, depression, and anxiety.

**Methods:**

In this cross-sectional survey, people with disabilities were recruited from sports clubs in southern Saudi Arabia. Club presidents were contacted via email or social media messaging services to access their member lists and identify potential participants. The sample comprised 205 participants who met the inclusion criteria and provided informed consent (age 18–40 years, mean = 29.8 years, standard deviation = 3.82 years). Participants received an email with a link to a Google Form containing information about the study and the questionnaire, which included the Social Inclusion Questionnaire for People with Disabilities, Mental Health Continuum - Short Form, Centre for Epidemiological Studies Depression Scale-10, and Generalized Anxiety Disorder-7. Data were analyzed using SPSS version 20. Cronbach’s alpha was used to assess the reliability of the study instruments. In addition to descriptive statistics, bivariate analyses (*t*-tests or one-way analysis of variance, as appropriate) were conducted to assess group differences. Pearson’s correlation coefficient and hierarchical regression analysis were used to examine the association between social inclusion and mental health outcomes (controlling for age, gender, and type of disability in regression analysis).

**Results:**

Slightly over half of the participants (53.65%) reported moderate to high social inclusion scores, while 46.34% had low inclusion scores. Social inclusion was positively correlated with overall mental health it was identified a significant predictor of mental health in the present sample.

**Conclusion:**

Present findings suggest that, among people with disabilities who are engaged in sports clubs, social inclusion was associated with better overall mental health and lower incidence of anxiety and depression. These results suggest the potential for sports clubs to become facilities that prevent the exclusion of people with disabilities and to support their physical, mental, and social well-being.

## 1 Introduction

People with disabilities experience psychological distress almost five times more frequently compared to non-disabled individuals (Nichols and Taylor, 2015; Waardenburg and Nagel, 2019). In 2018, an estimated 17.4 million (32.9%) of people with disabilities experienced recurrent psychological distress, which was in turn associated with poor health behaviors, increased use of health services, mental disorders, chronic diseases, and restrictions in daily life (Smith et al., 2018). Relatedly, several studies have confirmed that people with disabilities are at greater risk of experiencing mental health problems than are their non-disabled peers (Forlenza and Stella, 2020; World Health Organization, 2022; Smith et al., 2022; Smith and Sparkes, 2019). In part, their mental health outcomes are related to the social exclusion they experience in everyday life. According to the Convention on the Rights of Persons with Disabilities (United Nations, 2006), “Persons with disabilities include those who have long-term physical, mental, intellectual or sensory impairments, which in interaction with various barriers, may hinder their full and effective participation in society on an equal basis with others.” Similarly, based on the terms used in the International Classification of Functioning, Disability, and Health (ICF), “disability” conveys impairment and/or functional or participation limitations (Kostanjsek, 2011). When popular definitions of disability underscore the inability of these individuals to participate actively in society, it is not surprising that social exclusion is a foundational element of the disability experience. Therefore, human rights instruments such as the *United Nations Convention on the Rights of Persons with Disability* (CRPD) highlight the concept of social participation and inclusion (United Nations, 2006). Discrimination, negative attitudes toward disability, social value placed on the capabilities of people with disabilities, and systemic factors preventing inclusion are some underlying reasons for the exclusion of people with disabilities (Gooding et al., 2017).

Social exclusion is frequently contrasted with social inclusion; the former involves the stigmatization or marginalization of specific groups due to factors such as socioeconomic status, race, gender, or handicap. To be socially included, people with disabilities must overcome significant social, economic, and political obstacles in order to actively participate in society (Koller and Stoddart, 2021). Social inclusion is a multifaceted and frequently misconstrued notion (Kastenholz et al., 2015). It encompasses the capacity to establish social connections, participate in community activities, indulge in recreational and playful activities, and enjoy equitable and comprehensive educational procedures within the classroom.

Research on social inclusion must consider that forms of exclusion are interconnected and thus the exclusion of a person or group in one area may lead to exclusion in other areas. The ultimate goal of social inclusion is to ensure that everyone is included in society, regardless of the presence of limiting factors (Pavlencu, 2022; Crawford et al., 2023). In addition, a variety of terms, including “social inclusion” and “social integration,” are used interchangeably in both research and policy documents, this can create confusion, but it also reflects the evolving nature of these concepts. As Baumgartner and Burns (2013) explain, “social inclusion” is more commonly used in European contexts, while “social integration” often appears in American research. However, both terms aim to measure similar social outcomes: belonging, acceptance, and participation in a society.

Social inclusion is essential for achieving total well-being and is a crucial aspect of becoming a respected and active part of the community (Battams, 2016; Manzoor and Vimarlund, 2017). Social inclusion is key to promoting mental health among those with disabilities (Soundy et al., 2015) (World Health Organization, 2022; Pourmotabbed et al., 2020). Participation in sports has been shown to promote social inclusion by providing individuals with disabilities opportunities to build social networks, enhance self-esteem, and develop social skills. The United Nations’ Convention on the Rights of Persons with Disabilities recommends that participation in sporting activities aids physical and mental well-being of people with disabilities (United Nations, 2022). Previous studies (e.g., Kızar et al., 2015; Rogers and Pilgrim, 2021; Pečnikar Oblak et al., 2023) have highlighted the positive impact of sports on the social inclusion of people with disabilities. Engagement in the social activity of sport has been reported to bolster the sense of self, social identity, and community participation of people with disabilities (Kamberidou et al., 2019; Svanelöv et al., 2020). Participation in sports has been reported to improve quality of life, aids stress coping, and enhances self-esteem and social skills (Farì, 2023; Jaarsma et al., 2014). In their qualitative case study on nine athletes with disabilities in Columbia, Alvarez and Ramírez (2018) reiterated that sport was an effective method to facilitate social participation and to improve the quality of life of people with disabilities.

There is growing evidence to suggest the benefits of a sports lifestyle on the mental health of people with disabilities. For example adult athletes with cerebral palsy have reported a positive effects on their quality of life and mental health (Rogers and Pilgrim, 2021; Sarkova et al., 2014; Saxena et al., 2014). Several studies have also reported the alleviation of symptoms of depression, anxiety, stress, and PTSD, while also affording people with disabilities confidence by mastering a new skill (Adamson et al., 2015; Groot et al., 2016; Hagiwara et al., 2017; Limone and Toto, 2022; Rosenbaum et al., 2014). Several studies have confirmed that sports help prevent or relieve symptoms of depression among people with disabilities (Elmose-Østerlund et al., 2019; Rohwerder, 2018; Thapa and Kumar, 2015; Weston, 2017) and those with neurological disorders (Declerck et al., 2021). Participation in individual and team sports has also been shown to be beneficial for physical, social, psychological, and cognitive health outcomes (Nichols and Taylor, 2015; Waardenburg and Nagel, 2019). In addition, sport has been reported to reduce anxiety in people diagnosed with an anxiety or stress-related illnesses (Lee et al., 2014).

Since sport contributes to social inclusion (Kızar et al., 2015), it is important to encourage people with disabilities to exercise and participate in sports clubs, which enhances their mental health. Sports clubs are key service providers that encourage and facilitate the involvement of people with disabilities (Corthouts et al., 2020; Waardenburg and Nage, 2019). Ultimately, sports clubs can be seen as tools for building social inclusion, contributing to both physical and psychological well-being, and to improving the overall quality of life of people with disabilities (Albrecht et al., 2019; Di Palma et al., 2016; Kamberidou et al., 2019; Kissow, 2015; Kiuppis, 2018; Piatt et al., 2018).

Despite the numerous benefits of sports participation for people with disabilities, significant barriers persist. Social and cultural stigma, along with prejudices against their involvement in sports, remain major obstacles (Lankhorst et al., 2015; Pochstein et al., 2023). This is particularly evident when compared to the broader population. While international declarations like the United Nations Standard Principles on the Equalization of Opportunities for People with Disabilities and the International Convention on the Rights of People with Disabilities (UNCRPD) (United Nations, 2006) advocate for inclusion in sports, the reality is starkly different. This discrepancy suggests the presence of complex barriers hindering their engagement. While social stigma and cultural attitudes undoubtedly play a role (Jaarsma et al., 2014; Shields et al., 2012), personal factors like self-confidence, motivation, and physical fitness also contribute to this low participation (Elmose-Østerlund et al., 2019). Additionally, structural barriers such as inadequate facilities, transportation limitations, and time constraints pose significant obstacles (Declerck et al., 2021). Furthermore, Elmose-Østerlund et al. (2019) highlight the lack of specialized trainers, insufficient public services, and limited involvement of social organizations, further complicating the landscape for people with disabilities seeking to participate in sports (Lee et al., 2014).

In this regard, Saudi Arabia has sought to strengthen its support for sports, especially for people with disabilities. For example, the Saudi Disability Sports Federation established in 1991, now called the Saudi Arabian Paralympic Committee, opened 12 training centers for people with disabilities. An additional 15 sports centers and five additional clubs for people with disabilities were subsequently opened. Finally, in 2013, centers for people with disabilities were transformed into sports clubs for people with disabilities. In 2021, the “Pride Program,” was organized by the Saudi Paralympic Committee, under the aegis of the Ministry of Sports. This initiative falls under the Quality of Life Program, aimed at the goals of Vision 2030 (S. P. Agency, 2022). The latter focusses on rehabilitating people with disabilities, discovering and developing their sports abilities, promoting social integration, improving their quality of life, physical and psychological health, enhancing their community participation in sports activities, and creating sports heroes from them at the local and international levels (Zahra et al., 2022; Alanazi, 2023).

Though the commitment of the government in improving social inclusion of people of disabilities through sports is evident in such initiatives, there is little real-world data on how such activities influence people with disabilities. In general, there is paucity of research on individuals with disabilities in SA (Abed et al., 2024). Al-Jadid (2013) attributes this to lack of clarity or consistency in definitions of disability and related variables, shame and negative attitudes associated with disability and/or having a relative with a disability, and most importantly, a lack of commitment or motivation toward the well-being of a small proportion of the population. However, as per 2016 census data, 1 in 30 individuals in Saudi Arabia live with disabilities (Bindawas and Vennu, 2018). More recent reports estimate that about people with disabilities comprise 7.2% of the population of Saudi Arabia (Unified National Platform, 2024; Khalek and Elsabbagh, 2022). Evidently, this is not a small number, and therefore, research focused on the lived experiences of this population is the need of the hour if we wish to engage in data-driven, evidence-based policy making. With the key role that sports play in social inclusion and mental health of people with disabilities, it seems necessary to examine if this association holds true in the Saudi context. This research need was confirmed by Youngson et al. (2023), which found a scarcity of studies related to social inclusion and its role in promoting mental health among people with disabilities in Arab societies, especially Saudi Arabia. Accordingly, as a preliminary step in this direction, the present study aimed to fill this research gap by assessing the level of social inclusion experienced by people with disabilities who participated in sports clubs. Furthermore, it sought to examine the association between social inclusion and mental health outcomes in this population, while controlling for the influence of personal factors such as gender, age, and type of disability, which are intricately connected with the disability experience as well as with engagement in sports.

## 2 Methodology

### 2.1 Design and setting

Considering the aims of the present study and its exploratory nature owing to the paucity of research in the Saudi context, it was decided to conduct a cross-sectional survey utilizing standardized tools to assess the key study variables. In Saudi Arabia, “sports clubs” are key service providers that offer sporting experiences for people with disability. Set up by the Saudi Arabian Paralympic Committee (formerly known as the Saudi Disability Sports Federation), these clubs utilize sports to offer rehabilitation services, as well as aid social integration and physical and mental health support for people with disabilities through engagement in sporting activities. Therefore, these clubs offered the optimal setting to enable the researchers recruit an appropriate sample for the present study.

### 2.2 Participant recruitment

The target population comprised individuals with disabilities aged 18 or older, members of sports clubs offering disciplines such as athletics, volleyball, and swimming in southern Saudi Arabia. Due to geographical and resource constraints, the study focused on three such clubs.

Club presidents were contacted via email or social media messaging services, informing them of the study, and requesting their permission to access their member lists and contact participants for the study. Once lists were obtained, purposive sampling was used to identify people with disabilities who fit the following inclusion criteria: being aged over 18 years and being able to complete the study questionnaire online (by themselves or with assistance from a caregiver). “People with disabilities” was operationally defined as “Any person who has a complete or partial deficiency stably. The deficiency can be physical, sensory, mental, communicative, educational, or psychological capabilities,” as provided on the official website of the Saudi government (Unified National Platform, 2024). Note that this definition is in line with UN’s following established definition “those who have long-term physical, mental, intellectual, or sensory impairments which, in interaction with various barriers, may hinder their full and effective participation in society on an equal basis with others (United Nations, 2006). Thus, 205 eligible participants were selected. Research assistants approached potential participants within the clubs to provide study details and obtain informed consent. Data collection was primarily conducted online using a Google Form.

### 2.3 Data collection tools

Data were collected using an online questionnaire via Google Forms. The form included a section on the demographic characteristics of participants, including gender, age, type of disability, educational level, and frequency of going to the club. This was followed by four psychometric tools to measure social inclusion, overall mental health, depression, and anxiety.

#### 2.3.1 Social inclusion questionnaire for people with disabilities

The Social Inclusion Questionnaire for People with Disabilities (SIQ-PD) was employed to measure participants’ perceived level of social inclusion, which we define as ensuring all individuals, considered particularly at risk of social exclusion such as persons with disabilities, have opportunities to participate fully in society and enjoy equal rights. Developed by Albrecht et al. (2019), the SIQ-PD assesses social participation, acceptance, and support. Participants rated 14 items on a 5-point Likert scale (1 = strongly disagree, 5 = strongly agree). Scores range from 14 to 70, with higher scores indicating greater perceived social inclusion. Social inclusion levels were categorized as low, moderate, or high based on established cut-off points: low for scores below 2.33, moderate for scores between 2.34 and 3.67, and high for scores above 3.68.

Previous studies by Pierre et al. (2022) and Al Harthy et al. (2024) demonstrated the questionnaire’s good internal consistency (Cronbach’s alpha for the total scale score was 0.78, ranging from 0.59 to 0.71 for individual elements). Additionally, a half-stability coefficient of 0.84 further supports the SIQ-PD’s reliability and validity for use in this study.

#### 2.3.2 Mental health continuum—short form

The MHC–SF is a measure of mental health across three domains; subjective/emotional (3 items), psychological (6 items), and social well-being (5 items) (Keyeset al., 2008). The 14 items are rated on a 6-point Likert scale ranging from 0 (“never”) to 5 (“everyday”), with scores summed up to obtain total scores. Higher scores are indicative of higher levels of well-being. Keyes et al. (2008) found CIs over 0.80 for all domains and for the total score. In the current study, MHC-SF showed good viability and reliability, with good internal consistency (Cronbach’s *α* = 0.81). While specific categorical cut-points for classifying mental health levels are not established for the MHC-SF, the instrument provides a continuous measure of mental health.

#### 2.3.3 Centre for Epidemiological Studies Depression Scale (CES-D 10)

The CES-D 10 (Andresen et al., 1994) assesses symptoms of depression using 10 items across 3 domains: depressive affect (3 items), positive affect (2 items), and somatic symptoms (5 items). In each item, the frequency of experience of symptoms is rated on a 4-point scale ranging from 0 (rarely or none at all) to 3 (most or all of the time). Items 5 and 8, which pertain to positive affect, are reverse scored. Total scores range from 0 to 30, with a score of >10 used as a cut-off to indicate significant depressive symptoms. In the original study (Andresen et al., 1994), the scale showed good internal consistency reliability in the general population. The Arabic version of the scale has also been applied in many Saudi studies (Dewan et al., 2022; Nadim et al., 2016) with high reliability ratios (*p* < 0.005). In the current study, CES-D-10 showed good internal consistency (Cronbach’s α = 0.84).

#### 2.3.4 Generalized Anxiety Disorder-7

The GAD-7 is a 7-item short form developed by Spitzer et al. (2006) to measure the severity of generalized anxiety disorder symptoms. It utilizes the most prominent diagnostic features (diagnostic criteria A, B, C) of Generalized Anxiety Disorder, as defined in the Diagnostic and Statistical Manual of Mental Disorders, 5th Edition (DSM-V). Items are rated from 0 to 4, ranging from “not at all” to “almost every day.” The overall score ranges from 0 to 21, with scores higher than 10 considered to fall in the clinical range for diagnosing generalized anxiety disorder (Spitzer et al., 2006). The tool exhibited good internal consistency in the current study (Cronbach alpha = 0.84).

### 2.4 Data collection procedure

Data were collected from July to August 2023. Once the research assistants identified potential participants, they were contacted via email, with a link to a Google Form containing information about the study and the questionnaire described in section 2.3. Participants were informed that completion of the questionnaire would indicate their consent to voluntary participation. It was also clarified that all information obtained would remain strictly confidential, and that the data would only be used for scientific research purposes. Two reminders were sent to all potential participants via social media. Before the commencement of data collection, the study received ethical approval from Umm Al-Qura University, Kingdom of Saudi Arabia. In addition, all search procedures complied with the Declaration of Helsinki.

### 2.5 Data analysis

Data were analyzed using SPSS version 20. First, to assess the reliability of the study instruments, Cronbach’s alpha coefficients were calculated. Descriptive statistics including means and standard deviations, or frequencies and percentages were computed. Bivariate analyses were conducted using *t*-tests and one-way analysis of variance (ANOVA), as appropriate, to assess group differences, and Pearson’s correlation coefficient for correlation analysis. Finally, hierarchical regression analysis was used to examine the association between social inclusion and mental health outcomes while controlling for relevant demographic variables. Specifically, demographic variables that showed significant differences in the group comparisons outlined above were included as control variables in the regression analysis. For all analyses, significance was declared at *p* < 0.05.

## 3 Results

### 3.1 Demographic characteristics

The final sample comprised 205 people with disabilities aged 18–40 years (mean = 29.8 years years, standard deviation = 3.82 years). Majority of the participants were male (64.87%). In terms of disability types, motor disabilities were the most prevalent (33.65%), followed by sensory (29.26%), mental (20.97%), and other types (16.09%). Participants self-reported their primary disability type based on the provided categories. See Table 1 for data on all demographic characteristics assessed in the study questionnaire.

**Table 1:** Demographic characteristics of the study participants.

Demographic characteristics		*N*	%
Gender	Male	133	64.87%
	Female	72	35.13%
Age	18–23	56	27.31%
	24–30	87	42.43%
	31–40	62	30.24%
Disability types	Motor	69	33.65%
	Sensory	60	29.26%
	Mental	43	20.97%
	Other	33	16.09%

Regarding the disability classifications, these categories were determined by the researchers based on participant self-reports. Participants were asked to specify their primary type of disability. In cases of multiple disabling conditions, participants were requested to identify the condition they considered most impactful on their daily lives. This type of information was collected and categorized in the study’s methods section, specifically under the demographic data variables.

### 3.2 Social inclusion

Table 2 shows the levels of social inclusion in the current sample. Approximately 46% of participants reported low levels of social inclusion, while the remaining 54% reported moderate to high levels. Males reported significantly higher levels of social inclusion compared to females [*M* = 37.38, *SD* = 10.92 vs. *M* = 30.77, *SD* = 9.39, *t*(203) = 4.31, *p* < 0.001]. It is important to note that social inclusion, in this context, refers to the extent to which individuals with disabilities feel integrated and connected within their social environments, including participation in social activities, feeling valued, and having meaningful social interactions.

**Table 2:** Prevalence of social inclusion among people with disabilities.

Social inclusion scores	Social inclusion level	Frequency (*N* = 205)	Percentage
≤ 2.33	Low social inclusion	95	46.34%
2.34–3.67	Moderate social inclusion	83	40.48%
≥ 3.68	High social inclusion	27	13.17%

Tables 3, 4 shows the results of the t-test performed to compare social inclusion scores by gender. Male participants had higher social inclusion scores (*M* = 37.38, *SD* = 10.92) as compared to their female counterparts (*M* = 30.77, *SD* = 9.39).

**Table 3:** *t*-Test analysis of social inclusion based on gender.

Gender	*N*	*M*	*SD*	*t*
Male	133	37.38	10.92	4.31*
Female	72	30.77	9.39	

^*^Statistically significant at *p* < 0.05 level.

**Table 4:** ANOVA results examining social inclusion a questionnaire scores by gender, age, and type of disability among people with disabilities.

Source	Type III sum of squares	*df*	Mean square	*F*	Sig.
Corrected model	21990.964^a^	70	314.157	15.066	0.001
Intercept	53342.932	1	53342.932	2.5583	0.001
Gender	515.310	1	515.310	24.713	0.001
Age	149.674	2	74.837	3.589	0.030
Type of disability	325.047	3	108.349	5.196	0.002
Error	2794.138	134	20.852		
Total	280622.00	205			
Corrected total	24785.102	204			

^a^*R*^2^ = 0.887 (Adjusted *R*^2^ = 0.828).

Table 5 presents the results of the ANOVA test used to examine differences in the level of social inclusion based on age, type of disability, educational level, and frequency of attendance of a sports club. Significant differences were observed in groups based on age, and disability type, while educational level and frequency of attendance did not show significant differences in social inclusion scores.

**Table 5:** Scheffe test comparison of differences in social inclusion based on age and type of disability.

Variables		Mean difference (I-J)	Std. error	Sig.	95% CI	
					Lower bound	Upper bound
(I) Age	(J) Age					
18–23 years	24–30	−8.2007*	0.78232	0.001	−10.137	−6.2642
	years					
	31–40 years	−18.7408*	0.84183	0.001	−20.825	−16.657
24–30	18–23 years	8.2007*	0.78232	0.001	6.2642	10.1373
years						
	31–40 years	−10.5400*	0.75894	0.001	−12.419	−8.6614
31–40 years	24–30	18.7408*	0.84183	0.001	16.657	20.8246
	years					
	24–30	10.5400*	0.75894	0.001	8.6614	12.4187
	years					
(I) type of disability	(J) type of disability					
Physical disability	Intellectual disability	18.4031*	0.8872	0.001	15.8911	20.9151
	Visual disability	10.000*	0.80606	0.001	7.7178	12.2822
	Other disabilities	13.7879*	0.96647		11.0515	16.5243
Intellectual disability	Physical disability	−18.4031*	0.8872	0.001	−20.9151−	−15.8911−
	Visual disability	−8.4031*	0.91239	0.001	−10.9864−	−5.8198−
	Other disabilities	−4.6152*	1.05679		−7.6074−	−1.6231−
Visual disability	Physical disability	−10.000*	0.80606	0.001	−12.2822−	−7.7178−
	Intellectual disability	8.4031*	0.91239	0.001	5.8198	10.9864
	Other disabilities	3.7879*	0.98965	0.003	0.9858	6.5899
Other disabilities	Physical disability	−13.7879*	0.96647	0.001	−16.5243−	−11.0515−
	Intellectual disability	4.6152*	1.05679	0.001	1.6231	7.6074
	Visual disability	−3.7879*	0.98965	0.003	−6.5899−	−0.9858−

^*^The mean difference is significant at the 0.05 level.

The Scheffe test was used for post-hoc comparisons, to further assess the nature of differences in social inclusion scores based on age, and disability type. As evident from Table 5, social inclusion scores increased with age, with the 31-40-year-old participants exhibiting higher scores, followed by the 24-30-year-olds, and 18-23-year-olds, respectively. As for disability type, social inclusion scores were the highest among participants with physical disabilities, followed by those with visual, other, and intellectual disabilities, respectively.

### 3.3 Mental health outcomes

With reference to overall mental health, as assessed by the MHC-SF, the present sample reported a mean score of 35.98 points (*SD* = 1.62 points) on a possible score range of 0–70 points. The average score on the depression scale (CES-D 10) was 10.57 points (*SD* = 2.14 points) with a possible score ranging from 0 to 30 points. In addition, the average score on the anxiety scale (GAD-7) was 10.09 points (*SD* = 2.36 points), with a possible score ranging from 0 to 21 points.

### 3.4 Relationship between social inclusion and mental health outcomes

ANOVA was conducted to examine differences in social inclusion levels across mental health, depression, and anxiety scores. As shown in Table 6, individuals with high social inclusion exhibited higher mental health scores and lower depression and anxiety scores.

**Table 6:** Comparing Social inclusion level on mental health continuum, depression and anxiety scores (*N* = 205).

	Social inclusion	*N*	*M*	*SD*	*F*	*p*
Mental health continuum	Low	95	28.31	1.05	70.23	0.001
	Medium	83	40.23	1.18		
	High	27	50.62	1.30		
Depression	Low	95	11.45	2.85	13.05	0.001
	Medium	83	9.91	1.82		
	High	27	9.50	1.20		
Anxiety	Low	95	11.28	2.35	37.56	0.001
	Medium	83	9.53	1.86		
	High	27	7.53	1.79		

Furthermore, Pearson’s correlation analysis revealed a strong positive correlation (*r* = 0.66, *p* < 0.001) between social inclusion and overall mental health, indicating that higher social inclusion is associated with better mental well-being. A moderate negative correlation (*r* = −0.39, *p* < 0.001) was found between social inclusion and depression, suggesting a link between social connection and reduced depressive symptoms. Additionally, a strong negative correlation (*r* = −0.55, *p* < 0.001) emerged between social inclusion and anxiety, emphasizing the importance of social factors in mitigating anxiety.

While these correlations suggest a relationship between social inclusion and mental health outcomes, other factors may also influence this association. To account for potential confounders, hierarchical regression analysis was conducted with age, gender, and disability type as control variables. Results indicated that social inclusion significantly predicted mental health, depression, and anxiety, explaining 62.1% of the variance in social inclusion scores (*R*^2^ = 0.621, *F* = 54.17, *p* < 0.05).

Table 7 presents regression coefficients and significance levels for the relationship between social inclusion and mental health outcomes, controlling for demographic factors.

**Table 7:** Linear regression analysis of association between social inclusion and both mental health continuum, anxiety, depression, and demographic characteristics.

Variables	B	Std. error	Beta	*t*	*P*
Constant	39.92	4.15		9.6	0
Mental health continuum	0.4	0.044	0.458	9.29	0
Anxiety	−1.06	0.239	−0.239	−4.44	0
Depression	−0.545	0.224	−0.123	−2.43	0.016

Age, Gender, and type of disability were included as control variables.

Findings suggest that an increase in overall mental health scores was associated with a concurrent increase in social inclusion scores, while a decrease in anxiety and depression scores was associated with a concurrent increase in social inclusion scores. However, the low coefficient values indicate the presence of other factors in the association between social inclusion and mental health outcomes, which were not examined in the present study.

## 4 Discussion

This study assessed the level of social inclusion among people with disabilities who participated in sports clubs in southern Saudi Arabia, and examined its association with their mental health outcomes. The study had some interesting findings to offer. In the total sample, about half of the participants had low social inclusion scores, a surprising finding given that all participants were involved in sports clubs, activities often associated with enhanced social inclusion for people with disabilities (Kızar et al., 2015). People with disabilities often encounter obstacles and social exclusion, fueled by negative perceptions and discrimination rooted in disability-related stigma. This stigma can lead to exclusion from active participation in various life activities, including sports. This reality is supported by studies demonstrating lower sports participation rates among people with disabilities compared to their non-disabled counterparts (Albrecht et al., 2019; Kamberidou et al., 2019; Kiuppis, 2018; Klenk et al., 2019).

Our findings also suggest that demographic factors may contribute to differences in social inclusion, with women with disabilities facing potential double discrimination based on gender and disability. The data revealed higher social inclusion scores among males as compared to females. This disparity could be partly attributed to the sample composition, where male participants outnumbered females. However, it is crucial to acknowledge the broader societal context in Saudi Arabia. According to Alhumaid et al. (2022), cultural, religious, and gender issues exacerbate the barriers faced by female athletes with disabilities in Saudi Arabia. For instance, traditional gender roles and societal expectations from women in Saudi Arabia may restrict females with disabilities from participating in sports clubs, limiting their opportunities for social interaction and inclusion with both disabled and non-disabled individuals (Jeanes et al., 2022; Almateg et al., 2022; Zahra et al., 2022). This social exclusion can have adverse consequences, contributing to isolation and perpetuating negative stereotypes. Supporting research indicates that 93% of women with disabilities in Saudi Arabia do not participate in sports, and female athletes with disabilities represent only a third of the national contingent in international competitions (Almateg et al., 2022).

By fostering opportunities for women with disabilities to compete and showcase their athletic capabilities, sports can play a crucial role in challenging gender stereotypes and dismantling negative perceptions associated with this demographic.

Furthermore, the present findings revealed a higher level of social inclusion among individuals with motor disabilities as compared to those with other types of disabilities, such as intellectual or sensory impairments. This disparity may stem from the limitations faced by individuals with severe disabilities in actively participating in sports. Supporting this notion, Linz and Sturm (2016) highlighted the lower social inclusion levels experienced by individuals with severe disabilities due to challenges in developing soft skills. Additionally, other disability types, such as sensory and intellectual, may require specialized training programs that are often scarce, coupled with limited facilities and services specifically tailored to their needs (DePauw and Gavron, 2005; Shields et al., 2012).

When examining the lower levels of social inclusion observed in the overall sample as well as specific groups such as women, younger individuals, and those with visual, intellectual and other types of disabilities, it is important to acknowledge that the present study did not explore other impinging factors and barriers. For instance, the club’s functioning and hierarchical structure, inadequate support and incentives for people with disabilities, low perceived importance of the club participation by people with disabilities and/or their family, weak sense of belonging within the club as a cohesive social group are some sociocultural factors. Additionally, structural and systemic constraints pertaining to the status of individuals with disabilities owing to ableism and compulsory ablebodiedness, focus on team and competitive sports that exclude some individuals, inadequate accessibility of sports facilities and equipment, and transportation challenges need to be acknowledged. Financial constraints and the high cost of participation can be significant barriers. Furthermore, trainers may lack specialized knowledge or training to effectively support people with disabilities (Jaarsma et al., 2014; Shields et al., 2012). Studies by Jeanes et al. (2022) suggest that the limited role of sports clubs in promoting social inclusion stems from a lack of clarity about their purpose in this area. Specifically, they point to the absence of strategic actions and clear policies aligning with government and sports federation initiatives aimed at disability inclusion.

By addressing these challenges, we can create more inclusive environments that empower people with disabilities to enjoy the physical, social, and psychological benefits of sports participation. Future studies could explore why people with disabilities continue to experience lower social inclusion despite participation in sports clubs. Another interesting finding of the present study was that social inclusion scores did not differ significantly based on frequency of attendance of sporting clubs. This finding is interesting, as it suggests that factors beyond the mere attendance of sports clubs, which affect the quality of their sporting experience, may influence the social inclusion they experience. For instance, based on differences in social inclusion by disability type, one could conjecture that the nature of involvement in different sports, and the quality of experience, would differ based on the nature of barriers and limitations placed by the type of disability. Unfortunately, the present study could not explore the lived experiences of the participants. Future studies with different designs, e.g., qualitative, comparative, or longitudinal studies, could further explore the nature of participation in sports clubs more in depth.

Referring to the relationship between social inclusion and mental health outcomes examined in this study, the results of the different bi-variate and multivariate analyses utilized together suggested that increase in overall mental health scores was associated with a concurrent increase in social inclusion scores, while a decrease in anxiety and depression scores was associated with a concurrent increase in social inclusion scores. In interpreting these results, however, we need to be mindful of the fact that the cross-sectional design, and the analyses conducted in this study do not determine causality or the bi-directionality of the association between the study variables. Nevertheless, the association can be explained by some indirect pathways involving social inclusion and mental health among people with disabilities. Firstly, social inclusion has been found to positively influence health-promoting behaviors in general and mental health in particular, as well as risk-related behaviors (Kızar et al., 2015; Saxena et al., 2014). Further, feeling included or the sense of belonging derived from social activities such as sports clubs supports the development of self-efficacy, self-esteem, and coping skills, and the lowering of depression and distress (Adamson et al., 2015; Groot et al., 2016; Hagiwara et al., 2017; Limone and Toto, 2022; Rosenbaum et al., 2014; Aitchison et al., 2022; Youngson et al., 2023). As such, engaging in physical activity in itself has mental health benefits such as improved self-efficacy, better stress coping, alleviation of depression and anxiety symptoms, and overall improvement in quality of life (Weston, 2017; Hammad et al., 2024; Thapa and Kumar, 2015; Elmose-Østerlund et al., 2019; Jaarsma et al., 2014). Thus, the complexity of this association is evident, and the present study just scratches the surface of the intricate relationship between social inclusion and mental health outcomes along with a multitude of other intervening factors.

While this study offers valuable insights, it is essential to acknowledge limitations that warrant consideration when interpreting and applying the results to practice, and in generalizing the findings to broader contexts. Firstly, the sample selection and data collection methods introduced selection bias. Utilizing social media recruitment limited access to only those with internet connectivity and digital literacy, potentially excluding a significant portion of the target population. While ensuring anonymity through online methods was a commendable effort, future studies may benefit from alternative approaches to reach a more representative sample. Secondly, the sample size (*n* = 205) might limit generalizability to the entire southern Saudi Arabia region or the national level. While power analysis justifies the sample size, future studies should aim for larger, more diverse samples to provide a more comprehensive picture. Conducting comparative studies with sports clubs in other regions would further enrich the understanding of the investigated variables. Another limitation in the present sample was the lack of a comparison group. While the results speak to the experiences of the current sample, comparing them with people with disabilities not participating in sports clubs or with non-disabled peers would provide greater clarity on the association between the study variables and may aid the identification of other intervening factors that the current study could not do. Finally, incorporating other individual, social, cultural, economic, and structural factors into future research holds significant potential for deriving more holistic and powerful insights. Overcoming these limitations will be central for future studies to reach a better understanding of the complex interplay between sports clubs, social inclusion, and mental health for people with disabilities. Despite these limitations, this study stands out as one of the few exploring this topic within the specific context of people with disabilities in Saudi Arabia, offering valuable groundwork for future research.

The selection of three specific sports clubs in southern Saudi Arabia for this study imposes limitations on the generalizability of the findings. While these clubs were chosen to represent a range of sports, it is possible that they do not fully capture the diversity of sports clubs available to people with disabilities across the country. Future research should consider a broader sample of clubs to enhance the representativity of the findings. Additionally, detailed information about the characteristics of the selected clubs, such as size, resources, and specific programs offered, would provide valuable context for interpreting the results.

Despite offering preliminary insight on the variables studied, we can extrapolate some significant recommendations for practice and policymaking. Regarding the social inclusion of people with disabilities, we need to be mindful that, though sport in itself is considered beneficial for the physical and mental health of all individuals, people with disabilities often engage in activities that are offered in specific disability sports clubs or training groups, which may exacerbate their experience of discrimination and exclusion (Collins and Kay, 2014). Individual (e.g., disability type, social attitudes toward specific disabilities, internalized oppression) and structural (e.g., infrastructure, systemic discrimination, cultural, religious and legal issues) barriers to social participation in sport are a real part of their lived experiences (Jaarsma et al., 2014; Pierre et al., 2022). Therefore, when utilizing sport clubs as an avenue for offering social inclusion, we need to focus on developing club policies and programs that ensure that exclusionary practices are not perpetuated unconsciously.

At the macro level, it is important to institute strong laws and policies for promoting non-discrimination. For instance, recently Saudi Arabia enacted a new law called as The Law on the Rights of Persons with Disabilities (SLRPD), which reiterates the government’s commitment to the rights of people with disabilities, ensuring equality across age, gender, and social status (Saudi Law on the Rights of Persons with Disabilities, 2023). In addition to strong protective laws, policy makers need to focus on increasing awareness about the lived experiences, challenges, and barriers experienced by people with disabilities in Saudi Arabia. Media campaigns, school education programs, partnerships with organizations, professional agencies, private companies, and non-governmental organizations could contribute toward such efforts. These initiatives need to be aligned to the goals of Vision 2030 that aim to achieve dignity, success, and well-being for all Saudi Arabian citizens (Abobaker, 2025; Nasser and Al-Gharaibeh, 2023).

## 5 Conclusion

The present paper investigated the level of social inclusion experienced by people with disabilities participating in sports clubs in southern Saudi Arabia. Additionally, it explored the association between social inclusion and mental health in this rarely-researched target population. This cross-sectional study was the first of its kind in this context. This study revealed interesting findings on the level of social inclusion in this sample, and the influence of socio-demographic factors on social inclusion. Additionally, it suggested that, among people with disabilities who are engaged in sports clubs, social inclusion was associated with better overall mental health and lower incidence of anxiety and depression. Albeit preliminary, these findings are a first step toward understanding the potential utility of sports clubs in facilitating both social inclusion and mental health outcomes in this population. The results of this study could motivate the government and professionals working with people with disabilities to focus on addressing various systemic, social, and cultural barriers to accessing and enjoying the physical, mental, and social benefits of sports clubs in Saudi Arabia. Studies such as this could also be used to advocate for individuals with disabilities by using this evidence to convince their caregivers, sports coaches, physical therapists and other interested parties of the benefits of sports participation for this population.

## Data Availability

The original contributions presented in the study are included in the article/supplementary material, further inquiries can be directed to the corresponding author.
